# Application of a Halophilic Consortium for Saline Phenol Wastewater Treatment and Bioelectricity Generation in Microbial Fuel Cells: An AI-Driven Evaluation

**DOI:** 10.3390/biotech15040086

**Published:** 2026-10-01

**Authors:** Rawan Mohammed Alarishi, Arulazhagan Pugazhendi, Dinesh Kumar Mathew, Babajan Banaganapalli, Kowsalya Palanisamy, Tabreaz Basrur Zainula, Jeyakumar Dhavamani

**Affiliations:** 1 Department of Marine Biology, Faculty of Marine Sciences, King Abdulaziz University, Jeddah 21589, Saudi Arabia; ralarishi0005@stu.kau.edu.sa; 2 Center of Excellence in Environmental Studies, King Abdulaziz University, Jeddah 21589, Saudi Arabia; 3 Department of Civil Engineering, Saveetha School of Engineering, Saveetha Institute of Medical and Technical Sciences (SIMATS), Chennai 602105, Tamil Nadu, India; daviddinesh2009@gmail.com; 4 Department of Genetic Medicine, Faculty of Medicine, King Abdulaziz University, Jeddah 21589, Saudi Arabia; bbabajan@kau.edu.sa; 5 Princess Al-Jawhara Al-Ibrahim Center of Excellence in Research of Hereditary Disorders, King Abdulaziz University, Jeddah 21589, Saudi Arabia; 6 Department of Energy Science, Sung Kyun Kwan University, Suwon 440 746, Republic of Korea; kowsinst@gmail.com; 7 Department of Environmental Protection and Control, Global Environmental Management Services (REVIVA), Jeddah 21514, Saudi Arabia; tabreaz@yahoo.com; 8 Department of Technical Services (Environment), Global Environmental Management Services (REVIVA), Jeddah 21514, Saudi Arabia; jeyakumar@reviva.sa

**Keywords:** wastewater treatment, microbial fuel cell, halophiles, biofilm, energy, artificial intelligence, phenol biodegradation

## Abstract

High organic pollutant load and salinity pose major challenges for the biological treatment of industrial wastewater. This study evaluated an air-cathode microbial fuel cell (AC-MFC) inoculated with halophilic microorganisms for the simultaneous treatment of phenol-contaminated saline industrial wastewater and bioelectricity generation. The AC-MFC was operated at organic loads (OLs) of 1.2, 2.5, 3.2, 4.8, 6.2 and 8.6 g chemical oxygen demand (COD)/L, with optimum performance at 6.2 g COD/L. In optimal conditions, the system achieved a maximum voltage of 803 mV, power density of 460.6 mW/m^2^, current density of 573.6 mA/m^2^, and total and soluble COD removal efficiencies of 90% and 89%, respectively. Complete phenol degradation was achieved within 8 days. High-throughput sequencing revealed dominant halophilic taxa, including *Ochrobactrum*, *Marinobacter*, *Bacillus* and *Stenotrophomonas*, which may contribute to pollutant degradation and extracellular electron transfer. A Random Forest regression model implemented using Python/scikit-learn was further developed using OL and operational time as input variables to predict treatment and electrochemical performance. The model identified 6.2 gCOD/L as the optimum OL, consistent with the experimental results. Overall, inoculation of halophilic consortium coupled with AC-MFC technology demonstrates potential for simultaneous treatment of saline phenolic wastewater and bioelectricity generation, while artificial intelligence provides a useful tool for process optimization.

## 1. Introduction

In recent times, there has been growing interest in utilizing industrial wastewater to produce valuable products or energy, aiming to offset the high costs associated with wastewater treatment methods. Industrial wastewater often contains various phenol derivatives, heavy metals, and high salinity levels, presenting significant challenges for effective treatment. Industrial wastewater from petroleum refining, petrochemical processing, coal/coking, pharmaceutical manufacturing, textile, leather, and other chemical industries can contain phenolic compounds together with elevated concentrations of dissolved salts. Phenolic compounds are environmentally important contaminants because of their toxicity and persistence, while saline effluents can impose additional stress on receiving environments and biological treatment systems. Petroleum refinery and related industrial wastewaters may contain phenols together with other organic and inorganic contaminants, making treatment particularly challenging. Phenol-containing saline wastewater is, therefore, an important target for biological treatment because simultaneous phenol toxicity and salinity can restrict the activity of conventional microorganisms [1,2,3].

The concentrations of phenol and salinity vary substantially according to the industrial sector, production process, and wastewater stream. Typical petroleum refinery wastewater has been reported to contain phenol concentrations of approximately 20–200 mg/L [4]; however, substantially higher concentrations have been reported in specific process streams. Kusworo et al. [5] reported phenolic concentrations of approximately 950 mg/L in PRWW and an average phenol concentration of 960 mg/L in residual fluid catalytic cracking (RFCC) wastewater from a refinery in Indonesia, demonstrating that high-strength phenolic refinery wastewater can contain phenol concentrations approaching 1 g/L. Refinery spent-caustic streams are characterized by high alkalinity, organic loading, and elevated dissolved-salt concentrations. Reported salt concentrations in spent-caustic wastewater range from approximately 35.6 to 89 g/L, depending on the refinery process and operating conditions [6,7].

Technology employed in the treatment of industrial wastewater such as physical, chemical and biological has been unable to successfully accomplish pollutant removal and meet the stringent effluent discharge limits from the international environmental protection agencies. Among the technologies employed, biological treatment plays vital role in phenol removal under saline conditions. For example, *Halomonas* sp. Strain PH2-2 degraded up to 1100 mg/L phenol and remained capable of phenol removal in medium containing 18% NaCl, although removal efficiency decreased at higher salinity [8]. Other studies have demonstrated phenol degradation by halotolerant microorganisms under elevated salt concentrations [2,3]. These observations demonstrate that saline phenolic wastewater can present simultaneous organic toxicity and osmotic stress and, therefore, requires microorganisms with appropriate physiological adaptation.

High salinity imposes osmotic stress on microbial cells because the increased extracellular solute concentration creates an osmotic gradient across the cell membrane. This can cause cellular water loss, disturb membrane and protein functions, and reduce microbial growth and metabolic activity. Consequently, microorganisms that are not adapted to saline conditions may exhibit reduced substrate utilization and pollutant degradation. The biodegradation of phenol under saline conditions is strongly influenced by salinity, pH, temperature, phenol concentration, and microbial adaptation [3]. In contrast, halophilic and halotolerant microorganisms possess physiological mechanisms that allow them to maintain cellular homeostasis under elevated salt concentrations, including regulation of intracellular ions and accumulation of compatible solutes [9,10]. Recent reviews further indicate that high salinity can decrease microbial diversity, abundance, and activity in biological wastewater treatment systems, while microbial enhancement approaches can alleviate salt-induced inhibition [10]. Phenol biodegradation by salt-adapted microorganisms has been demonstrated in a range of saline environments. Phenol-degrading microorganisms belonging to genera, such as *Halomonas*, *Bacillus*, *Pseudomonas*, *Rhodococcus* and other bacterial groups, have been reported under saline or hypersaline conditions [3]. However, maintaining sufficient abundance and activity of functional microorganisms within an engineered treatment system remains an important challenge.

The application of a halophilic consortium is an effective biological approach for treating saline wastewater containing recalcitrant pollutants. The effectiveness of the consortium in wastewater treatment depends on factors, such as consortium composition, acclimation, microbial survival and activity, substrate availability, operating conditions, and interactions among microorganisms within the treatment system [11]. For saline wastewater, the selection of microorganisms that are both salt-tolerant and capable of degrading the target pollutant is particularly important.

The relevance of employing halophilic consortium for saline wastewater treatment is supported by previous microbial fuel cell (MFC) research. Pugazhendi et al. [12] demonstrated that halophilic consortium in an air-cathode MFC operated under saline conditions resulted in substantial chemical oxygen demand (COD) and total suspended solids (TSS) removal, complete phenol removal and simultaneous bioelectricity generation. Their study also demonstrated the dominance of halophilic electrogenic bacteria, including *Ochrobactrum*, *Marinobacter*, *Bacillus* and *Rhodococcus*, under different organic loads [12]. These findings indicate that inoculation of halophilic consortium can simultaneously address salt stress, organic pollutant degradation, and electroactive activity in saline MFC systems.

MFCs provide an alternative biological treatment platform in which microorganisms oxidize biodegradable organic substrates at the anode and transfer electrons through an external circuit, thereby generating electricity while reducing the organic load of wastewater [13,14]. Air-cathode MFCs are particularly attractive because their single-chamber configuration uses oxygen from air at the cathode and can reduce system complexity and operational requirements [14,15,16]. Under saline conditions, increased ionic strength can enhance solution conductivity and reduce ohmic resistance, thereby potentially improving electron transfer and electrochemical performance; however, excessive salinity can simultaneously inhibit microorganisms that lack salt tolerance [16,17]. Therefore, the use of halophilic or halotolerant electroactive microorganisms is relevant for maintaining biological activity and electron generation in saline MFCs.

Organic loading is an important operating parameter in MFCs because it determines substrate availability to electroactive microorganisms and influences pollutant removal, electron generation, current production and power output. Increasing organic loading can enhance electrochemical activity up to an optimum level, whereas excessive loading may cause substrate inhibition, mass-transfer limitations, accumulation of inhibitory compounds, or increased internal losses [18]. Therefore, optimization of organic loading is necessary to balance wastewater treatment and bioelectricity generation. In addition to total COD and soluble COD, TSS provides complementary information on the suspended particulate fraction of wastewater and can be used to evaluate the reduction in suspended solids during treatment. TSS removal is not interpreted as a direct measure of phenol biodegradation; rather, it complements phenol, COD, and electrochemical measurements by providing an additional indicator of overall treatment performance. The relevance of TSS measurement is also supported by previous saline AC-MFC research in which TSS removal varied with organic loading and electrochemical performance [12].

Despite increasing interest in halophilic microorganisms and MFC-based treatment, further investigation is required into the simultaneous treatment of phenol-containing saline industrial wastewater and bioelectricity generation using halophilic consortium. In particular, the relationship between organic loading, pollutant removal, suspended solids removal, and electrochemical performance can be nonlinear and difficult to optimize using conventional approaches. Artificial intelligence (AI)-based approaches can provide complementary tools for identifying relationships among operational variables and treatment responses and for evaluating process performance. Therefore, the present study investigated an air-cathode MFC inoculated with halophilic bacterial consortium for the treatment of phenol-containing saline industrial wastewater under different organic loads. Treatment performance was evaluated using total chemical oxygen demand (TCOD), soluble chemical oxygen demand (SCOD), TSS, phenol removal, and bioelectricity generation, while AI was applied to evaluate the relationship between organic loading and treatment efficiency and corresponding electrochemical performance.

## 2. Materials and Methods

### 2.1. Sample Collection and Wastewater Characterization

Phenol-contaminated saline industrial wastewater (PSIWW) was collected in 5 L fresh bottle from petroleum/plastic-producing industry, Yanbu, Saudi Arabia. The samples were stored in refrigerator at 4 °C for future use.

Physicochemical analyses of raw PISWW results were pH—10.5, total dissolved solids—193.2 g/L, total suspended solids (TSS)—11,380 mg/L, total chemical oxygen demand—38,400 mg/L and salinity—18.4%.

### 2.2. Experimental Design and Operational Conditions

**Figure 1:**
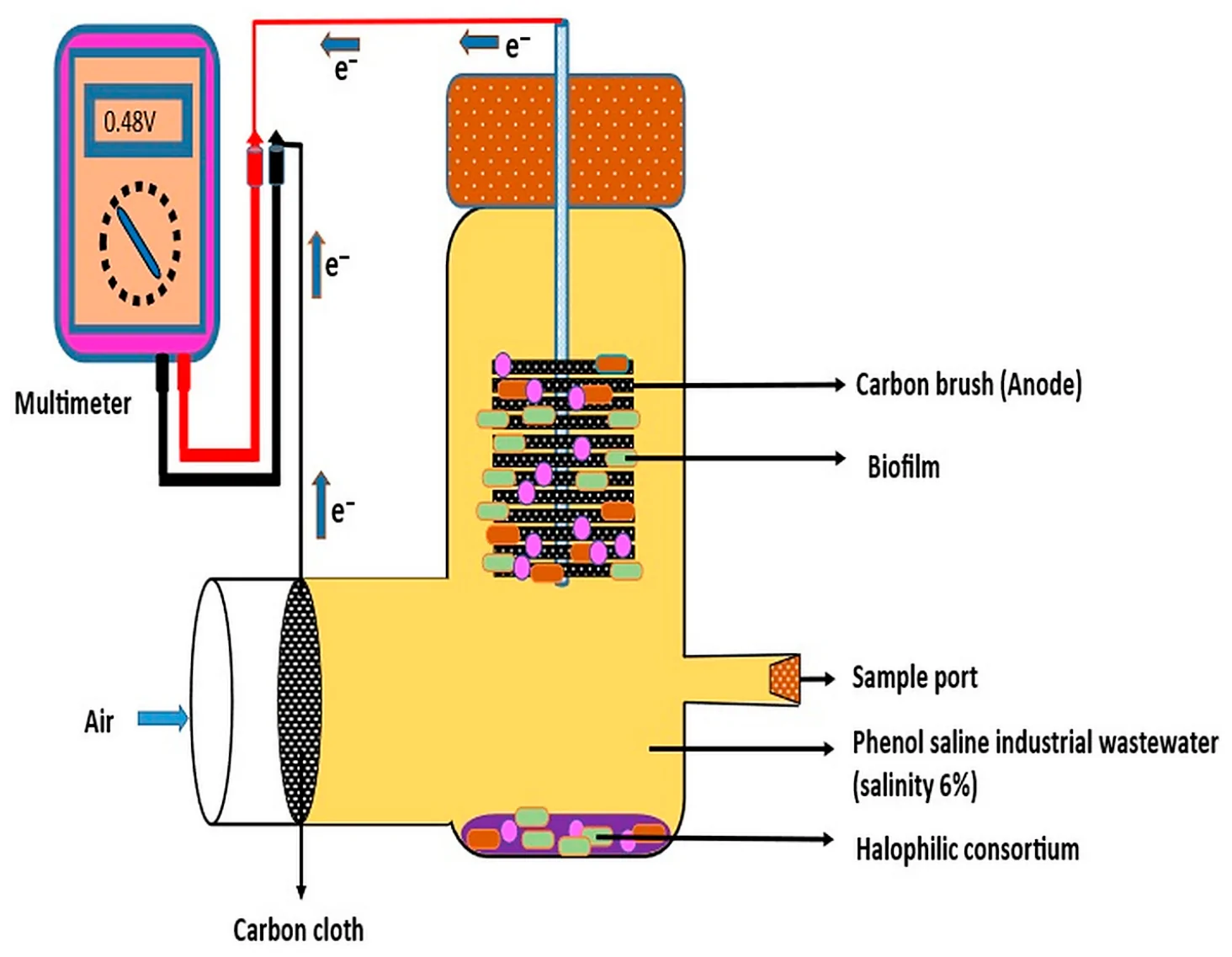
Schematic representation of air-cathode microbial fuel cell.

Air-cathode MFC was fabricated with carbon brush as anode and cloth as cathode. The total volume of the reactor was 330 mL. Digital multimeter (Greenlee DM-45, Rockford, IL, USA) connected with anode and cathode was used to record the electricity generated during the operation of AC-MFC (Figure 1). Organic loads (OLs), such as 1.2, 2.5, 3.2, 4.8, 6.2, and 8.6 gCOD/L, were applied in the reactor with 6% salinity at 30 °C. These values represent COD concentration in the wastewater and are, therefore, referred to as organic load (OL; g COD/L), rather than organic loading rate. The collected industrial wastewater had an initial COD concentration of 38.4 g COD L^−1^ and salinity of 18.4%. Prior to the AC-MFC experiments, the wastewater was diluted with ultrapure Type 1 water (18.2 MΩ·cm resistivity at 25 °C) produced using a Milli-Q (Milli Q Direct 8, Molsheim, France) water purification system to obtain the required organic loads (OLs) of 1.2, 2.5, 3.2, 4.8, 6.2, and 8.6 g COD/L. The corresponding wastewater-to-deionized water dilution ratios were approximately 1:31, 1:14.4, 1:11, 1:7, 1:5.2, and 1:3.5, respectively. Following dilution, the salinity of each OL was adjusted to 6% by adding NaCl to compensate for the variation in salinity resulting from dilution. Thus, all experimental conditions were maintained at approximately 6% salinity while varying the organic load. The corresponding initial phenol concentrations at the six OL conditions were 210, 390, 480, 560, 610, and 720 mg/L, respectively. The same AC-MFC reactor was used sequentially for the different OL conditions rather than using independent reactors for each condition. Each OL condition was maintained for 16 days. The subsequent OL condition was initiated after no further improvement in organic matter removal efficiency was observed during the preceding stage. Therefore, the experimental design represents a sequential single-reactor study, in which temporal and stage-related effects cannot be completely separated from the effect of OL. Experimental measurements were conducted in duplicate, and corresponding samples were analyzed in triplicate. The analytical replicates were used to assess measurement reproducibility and are not equivalent to independent biological reactor replicates. This distinction is considered when interpreting the statistical analyses and the machine learning results.

#### Inoculation of Halophilic Consortium

Inoculation of halophilic consortium (30 mL) enriched from desalination plant was introduced into the MFC reactor. Industrial wastewater with high salinity is not potently treated by the non-halophilic bacterial strain. Salinity and phenol concentration highly influence the growth and mineralization activity of the bacterial strains. To overcome this problem, halophiles, which actively perform organic pollutant mineralization under saline conditions, were employed. Halophilic consortium enriched from desalination brine water was inoculated in AC-MFC possess: *Ochrobactrum* (PV689948), *Marinobacter*, (PV689949), *Bacillus*, (PV689950), *Stenotrophomonas*, (PV689951), *Rhodococcus*, (PV689952), *Pseudomonas*, (PV689953), *Acinetobacter*, (PV689954), *Sphingomonas*, (PV689955), *Sedimentibacter*, (PV689956), *Alicyclobacillus* (PV689957) and *Microbacterium* (PV689958). The raw industrial wastewater was subjected to DNA extraction and high-throughput sequencing analysis, which revealed absence of a detectable bacterial community. Also, no other external microbial source was introduced into the reactor. Therefore, the halophilic consortium was the only bacterial inoculum intentionally introduced into the AC-MFC. The bacterial community detected during reactor operation consequently originated from the inoculated halophilic consortium. The reactor was operated in fed batch mode. Reactors operated without the halophilic consortium acted as the control, which exhibited neither organic removal nor bioelectricity generation.

### 2.3. Analytical Procedures

#### 2.3.1. COD and TSS Analysis

Total COD (chemical oxygen demand), soluble COD and total suspended solids (TSS) were analyzed using standard methods by APHA [19]. For soluble chemical oxygen demand (SCOD) determination, wastewater samples were filtered using Merck Millex hydrophilic PTFE syringe filters (0.45 µm pore size; Merck, Darmstadt, Germany) to remove suspended particulate matter. The resulting filtrate was subsequently analyzed for COD according to the standard method described by APHA [19]. The treatment performance was evaluated using TCOD, SCOD, TSS and phenol concentration. TCOD and SCOD were used to quantify the removal of total and soluble organic loads, respectively, while phenol removal was determined to specifically assess degradation of the target contaminant. TSS removal was additionally evaluated to assess the reduction in suspended matter. These parameters were selected to comprehensively characterize organic load and phenol removal from the saline industrial wastewater. Although biochemical oxygen demand (BOD) and total organic carbon (TOC) are valuable complementary indicators of organic matter removal, the combination of TCOD, SCOD, TSS, and phenol degradation provided an appropriate assessment of the overall organic removal efficiency in the MFC system.

#### 2.3.2. Total Phenol Analysis

Total phenol was analyzed via high-performance liquid chromatography (Agilent 1260, Waldbronn, Germany) equipped with ZORBAX Eclipse Plus C18 reversed-phase analytical column (4.6 × 150 mm, 5 µm particle size, 95 Å pore size; Agilent Technologies, Newport, DE, USA) as stationary phase. Acetonitrile (ACN) + 1% acetic acid was used as the mobile phase, and phenol was detected using a UV detector at 284 nm, as described by Pugazhendi et al. [12]. All experiments were performed in duplicate, and the corresponding samples were analyzed in triplicate to ensure the reproducibility and reliability of the experimental results.

### 2.4. Evaluation of Bioelectricity Generation

To evaluate current and power generation in a single-chamber air-cathode MFC at various organic loads, the following equations were applied:

**I = E/R** (1)

**P = Ι × E** (2)

where:

Ι = Current (A)

E = Voltage (V)

R = External resistance (Ω)

P = Power (W)

Power density and current density were calculated based on the projected geometric surface area of the carbon-brush anode (0.014 m^2^) [20]. After a 16-day acclimatization period under open-circuit conditions, each organic loading (OL) condition was evaluated under closed-circuit conditions by sequentially applying external resistances ranging from 100 to 16,000 Ω to obtain the polarization curves [21]. At each external resistance, the MFC was allowed to equilibrate for 20 min, after which the voltage was recorded under steady-state conditions. Current was calculated using Ohm’s law, and the corresponding current and power densities were calculated based on the projected geometric surface area of the anode. Each OL condition was operated for 16 days under closed-circuit conditions, during which voltage, current, and organic removal were monitored; no further improvement in organic removal or electricity production was observed after 16 days. The efficiency of coulomb recovery, or coulombic efficiency (CE), was determined by measuring the ratio of recovered coulombs to the total theoretical coulombs available from the substrate. This efficiency for each fed-batch cycle was computed using the formula below [12].

**CE = (8 × I × tb)/(F × V ΔCOD)** (3)

where:

8 = the theoretical COD equivalent of one mole of electrons (8 g COD mol^−1^ e^−^), based on the transfer of 4 mol of electrons per mole of O_2_.

I = Current (A)

tb = Time (s)

F = Faraday constant (96,500 C/mol-electrons)

V = Anode volume (L)

ΔCOD = Change in COD (g/L)

### 2.5. Estimation of Bacterial Profile

DNA isolation was performed in samples from the reactor (anode and sludge region) at different OLs. DNA analysis was performed on duplicate samples obtained at each operated OL. High-throughput sequencing methods revealed the bacterial strains’ percent occupancy in the reactor. Primers targeting the V4—-V5 region of the 16S rRNA gene (515—-532U 5′-GTGYCAGCMGCCGCGGTA-3′ and 909—-928U 5′-CCCCGYCAATTCMTTTRAGT-3′) were used, and sequencing was conducted at 65 °C for 30 cycles to ensure thorough amplification [22]. Further, the obtained sequences were assembled using Mothur software version 1.35.0, following a modified MiSeq standard protocol. Additional steps included chimera checking with UCHIME and pre-clustering based on sequence length. Taxonomic alignment was achieved using SILVA SSURef NR99 (Small Subunit Reference Non-Redundant 99% sequence identity database, Braunschweig, Germany), release 119, to analyze the microbial community structure, with bacterial genus classifications confirmed through BLAST (Basic Local Alignment Search Tool), version 2.17.0 [23]. Nucleotide sequences of the strains were submitted in NCBI (National Center for Biotechnology Information), and the respective accession number was obtained.

### 2.6. AI (Artificial Intelligence) Evaluation

A supervised machine learning framework was implemented to model nonlinear relationships between operational parameters and MFC performance in the treatment of PSIWW. Random Forest regression was selected for this task on the basis of its demonstrated robustness on small, tabular datasets and its capacity to model nonlinear interactions without requiring a pre-specified functional form [24], which is well suited to the nonlinear substrate loading and biofilm maturation dynamics characteristic of bioelectrochemical systems [8,25]. All models were implemented in Python (version 3.12.7) using the scikit-learn (Version 1.7.2) library [26]. The deployment of the AI framework was specifically necessary to overcome the inherent limitations of empirical trial and error in microbial fuel cell operation. Machine learning was integrated to: (1) quantitatively decouple the relative influence of substrate loading versus biofilm maturation time on performance via feature importance; (2) capture nonlinear substrate inhibition kinetics across continuous operational domains; and (3) mathematically determine the multi-objective Pareto optimum, balancing power generation and TCOD removal without subjective selection bias.

Two independent regression models were constructed. The first model predicted power density (mW/m^2^) using organic load (OL, gCOD/L) and operational day as input variables. The second model predicted total chemical oxygen demand (TCOD) removal efficiency (%) using OL stage (gCOD/L) and operational day as predictors. The electrical model was trained on a dataset of *n* = 54 observations, derived from six OL levels (1.2, 2.5, 3.2, 4.8, 6.2 and 8.6 gCOD/L), each monitored across nine timepoints (Day 0–16) per stage. The TCOD removal model was trained on *n* = 48 observations, reflecting seven to nine timepoints per OL stage in the treatment sampling schedule. Both input features, OL and operational day, were used directly without scaling, as Random Forest is insensitive to feature magnitude.

Each dataset was partitioned into a training set (80%) and a hold-out test set (20%) using random sampling (random_state = 42) to obtain an unbiased estimate of generalization performance. To further assess robustness given the limited sample size, 5-fold cross-validation (CV) was applied to the full dataset, yielding the mean and standard deviation of R^2^, mean absolute error (MAE), and root mean squared error (RMSE) across folds. The Random Forest models were configured with the following hyperparameters: n_estimators = 100, max_depth = None (fully grown trees, no maximum depth constraint), min_samples_split = 2, min_samples_leaf = 1, max_features = ‘sqrt’ (square root of total features), bootstrap = True, and random_state = 42 to ensure reproducibility. Overfitting was assessed by comparing training versus test R^2^; a large discrepancy would indicate poor generalization. To contextualize Random Forest performance, two baseline comparators were evaluated on the same train/test partition: (i) multiple linear regression and (ii) second-degree polynomial regression. Because experimental observations were acquired sequentially across discrete operating cycles of the same reactor, random splitting inevitably places observations from proximate timepoints of the same organic loading stage into both training and test partitions. Consequently, the reported metrics reflect the model’s intra-system interpolation fidelity rather than extrapolation to novel reactor architectures or operating regimes. Given the small number of distinct loading stages (*n* = 6), grouped leave-one-stage-out validation would constitute extreme out-of-domain extrapolation for decision-tree ensembles; this operational limitation should be considered when interpreting predictive metrics.

Model performance was evaluated using the coefficient of determination (R^2^ = 1 − [Σ(y_i_ − ŷ_i_)^2^/Σ(y_i_ − ȳ)^2^]), the mean absolute error (MAE = (1/n) Σ|y_i_ − ŷ_i_|), and the root mean squared error (RMSE = √[(1/n) Σ(y_i_ − ŷ_i_)^2^]). To integrate electrical and treatment performance into a unified decision metric, predicted outputs were normalized using min–max scaling (X_norm = (X − X_min)/(X_max − X_min)) and combined into a composite score (score = 0.5 × PD_norm + 0.5 × Removal_norm). The equal weighting of power density and TCOD removal reflects the equivalent operational priority assigned to energy recovery and wastewater treatment efficacy for sustainable MFC deployment [27,28]. Predicted power density was summarized per OL stage using the final timepoint (Day 16, present for all stages), and predicted TCOD removal was summarized using the mean of the last three available timepoints per stage, before ranking OL conditions by combined score.

### 2.7. Statistical Analysis

The experimental data were statistically analyzed using SPSS (Statistical Package for the Social Sciences), software version 17.0. A one-way univariate analysis of variance (ANOVA) was performed at a significance level of *p* < 0.05 to assess the validity, reproducibility, and statistical significance of the halophilic consortium’s performance in treating organic PSIWW.

## 3. Results and Discussion

### 3.1. Mineralization of Organic Substrate

#### 3.1.1. Total COD Removal Efficiency

**Figure 2:**
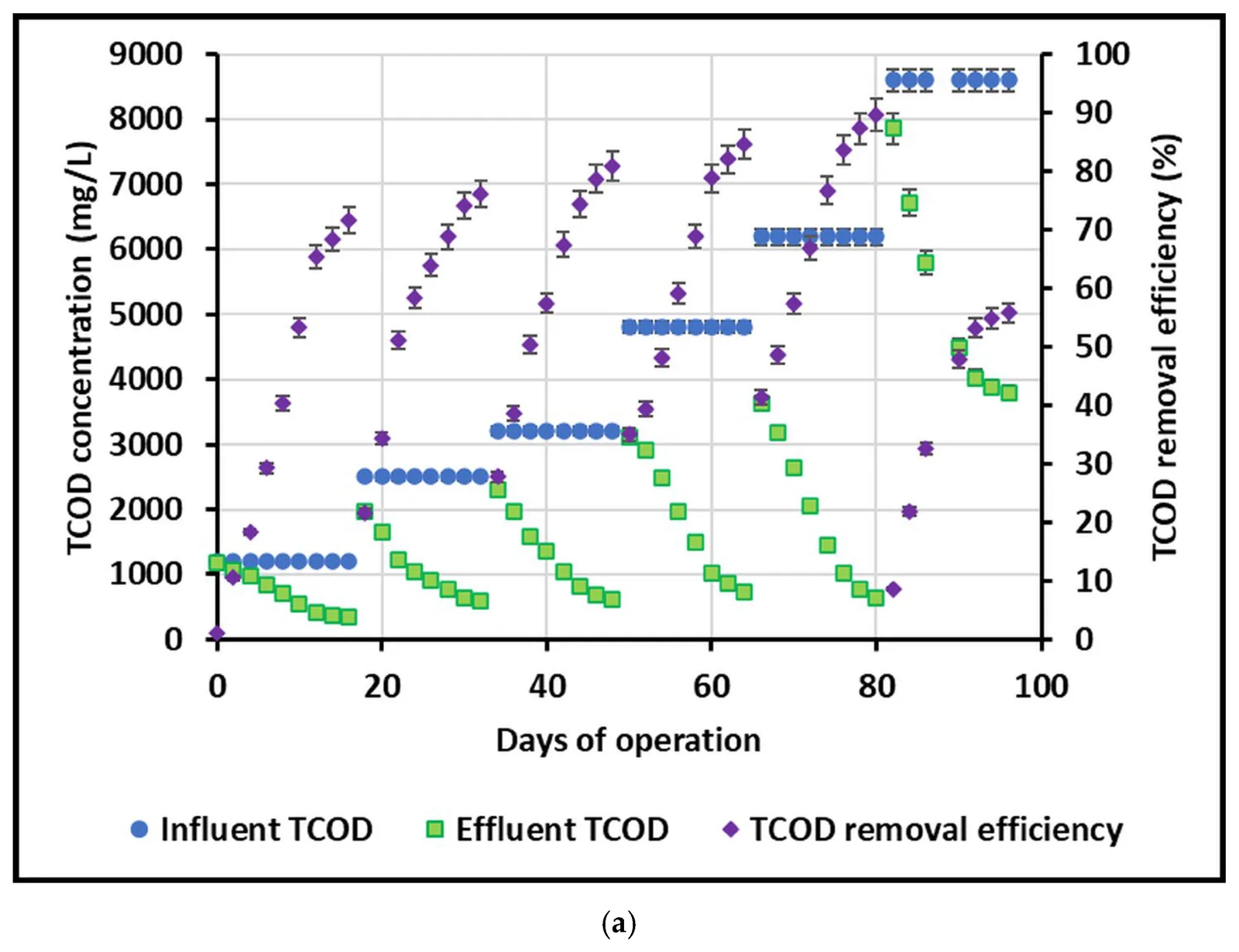

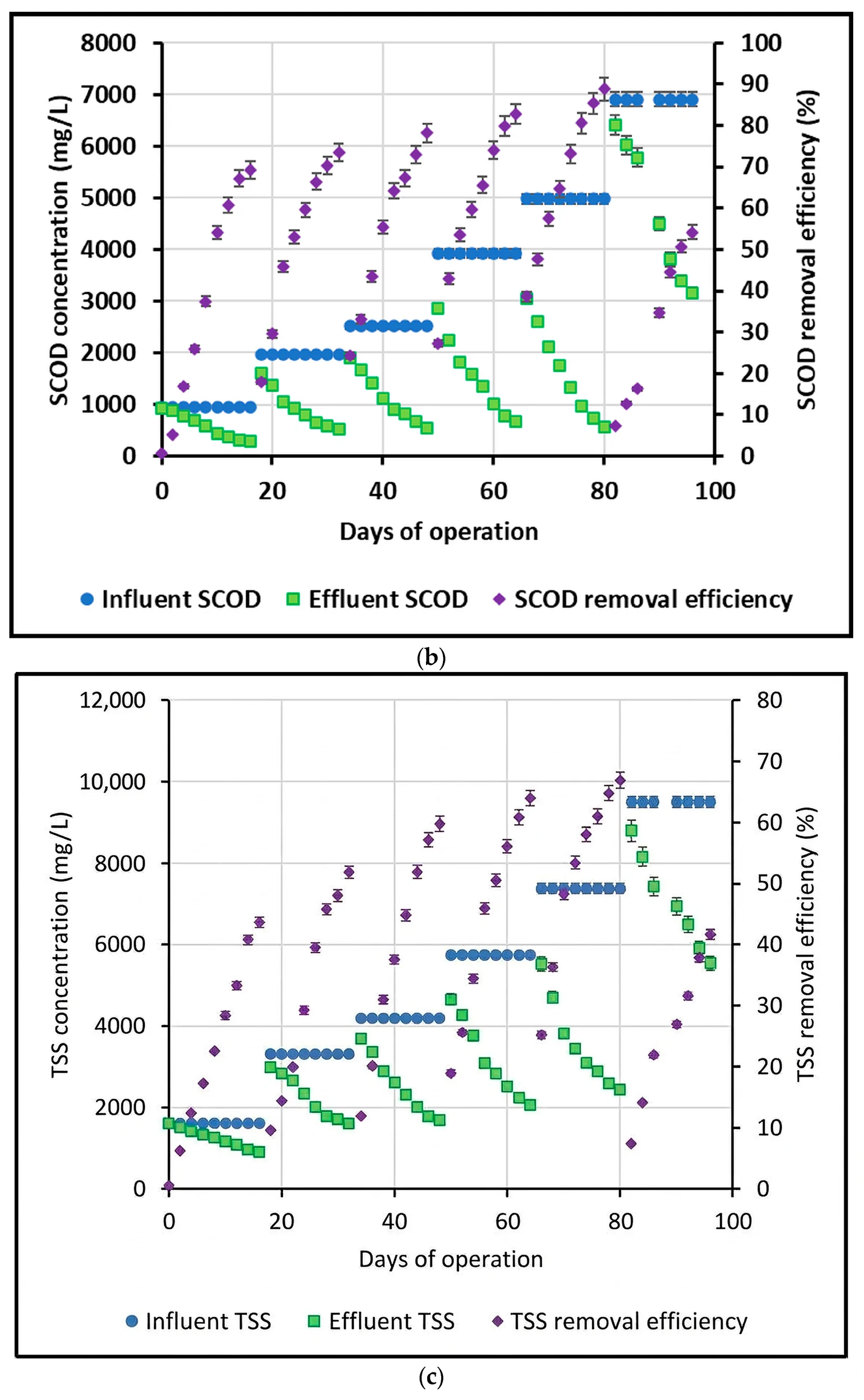
(**a**). TCOD removal efficiency. Data are presented as mean ± SD of triplicate analytical measurements from duplicate experimental runs. Statistical differences were determined by one-way ANOVA; (*p* < 0.001). (**b**). SCOD removal efficiency. Data are presented as mean ± SD of triplicate analytical measurements from duplicate experimental runs. Statistical differences were determined by one-way ANOVA; (*p* < 0.001). (**c**). TSS removal efficiency. Data are presented as mean ± SD of triplicate analytical measurements from duplicate experimental runs. Statistical differences were determined by one-way ANOVA; (*p* < 0.001).

Total COD removal was estimated in AC-MFC at various OLs (Figure 2a). The TCOD removals were 72%, 76%, 81%, and 85% at organic loads of 1.2, 2.5, 3.2, and 4.8 gCOD/L, respectively. The highest TCOD removal efficiency of 90% was achieved at an OL of 6.2 gCOD/L, while an OL of 8.6 gCOD/L resulted in a minimum TCOD removal efficiency of 56%. This reduction at the highest OL may be associated with excessive substrate loading, which can adversely affect microbial activity and substrate utilization in the anode chamber. Therefore, an OL of 6.2 gCOD/L was considered optimal based on the highest TCOD removal efficiency. Multiple studies have reported that MFCs achieve an efficiency of around 80–90% in removing COD [29]. The high amount of electrochemical activity has been found to improve with increasing organic load, but excessively high OL can lead to low activation and an increase in ohmic losses [8]. When the organic load reaches 6.2 gCOD/L, the microorganisms in the anode chamber begin fastidiously to mineralize the substrate, leading to a peak in system performance, with a TCOD removal efficiency of 90%. Grattieri and Minteer [25] reported firm attachment of bacterial strains in the carbon brush with high electrogenic efficiency accelerated the COD removal in MFC. However, further increasing the organic load to 8.6 gCOD/L resulted in a marked decline in TCOD removal efficiency to 56%. This reduction may be attributed to excessive substrate concentration, which can impose metabolic stress on the bacterial consortium and limit effective substrate utilization [30]. In addition, the high organic load may increase the accumulation of intermediate metabolites and create mass-transfer limitations within the anode biofilm, potentially restricting microbial activity and extracellular electron transfer. Consequently, excessive OL may negatively affect both substrate degradation and bioelectricity generation [31].

#### 3.1.2. Soluble COD Removal Efficiency

The SCOD removal efficiency shows a consistent upward trend with increasing OL, (Figure 2b), starting SCOD removal of 69% at 1.2 gCOD/L, rising to 73% at 2.5 gCOD/L, and further improving to 78% at 3.2 gCOD/L. The efficiency continues to climb, reaching 83% at 4.8 gCOD/L and peaking to 89% at 6.2 gCOD/L, indicating highly effective substrate degradation. Previous research studies clearly stated that a carbon brush with high surface area leads to thick biofilm formation, which accelerates the COD removal coupled with power generation [25,32]. However, as the OL increases to 8.6 gCOD/L, the SCOD removal efficiency sharply declines to 54%, suggesting a drop in system performance due to microbial overload or diminished processing capability at higher loads. This substantial decrease may be attributed to excessive substrate loading, which can impose metabolic stress on the bacterial consortium and reduce the efficiency of substrate utilization. At high organic loading, the increased substrate concentration may also cause accumulation of soluble metabolic intermediates and create mass-transfer limitations within the anode biofilm. Furthermore, the greater availability of the substrate relative to the electron transfer capacity of the anodic microbial community may reduce the efficiency of extracellular electron transfer and consequently limit the overall biodegradation process. Such effects can result in incomplete substrate utilization and a decline in SCOD removal at excessive loading conditions [20,33].

#### 3.1.3. Total SS Removal Efficiency

The TSS removal efficiency shows a gradual improvement with increasing organic load (OL) up to a point but experiences a decline at higher levels (Figure 2c). At 1.2 gCOD/L, the TSS removal efficiency starts at 44%, indicating moderate removal of total suspended solids at this low organic load. As the OL increases to 2.5 gCOD/L, the efficiency improves to 52% and continues to rise to 60% at 3.2 gCOD/L. At 4.8 gCOD/L, the TSS removal efficiency reaches 64%, demonstrating the system’s growing effectiveness. The maximum efficiency of 67% occurred at 6.2 gCOD/L, reflecting optimal performance in TSS removal. However, at 8.6 gCOD/L, the efficiency drops to 42%, suggesting that the system struggles with higher organic loads, likely due to microbial or filtration limitations, leading to reduced effectiveness in removing suspended solids.

### 3.2. Phenol Removal

**Figure 3:**
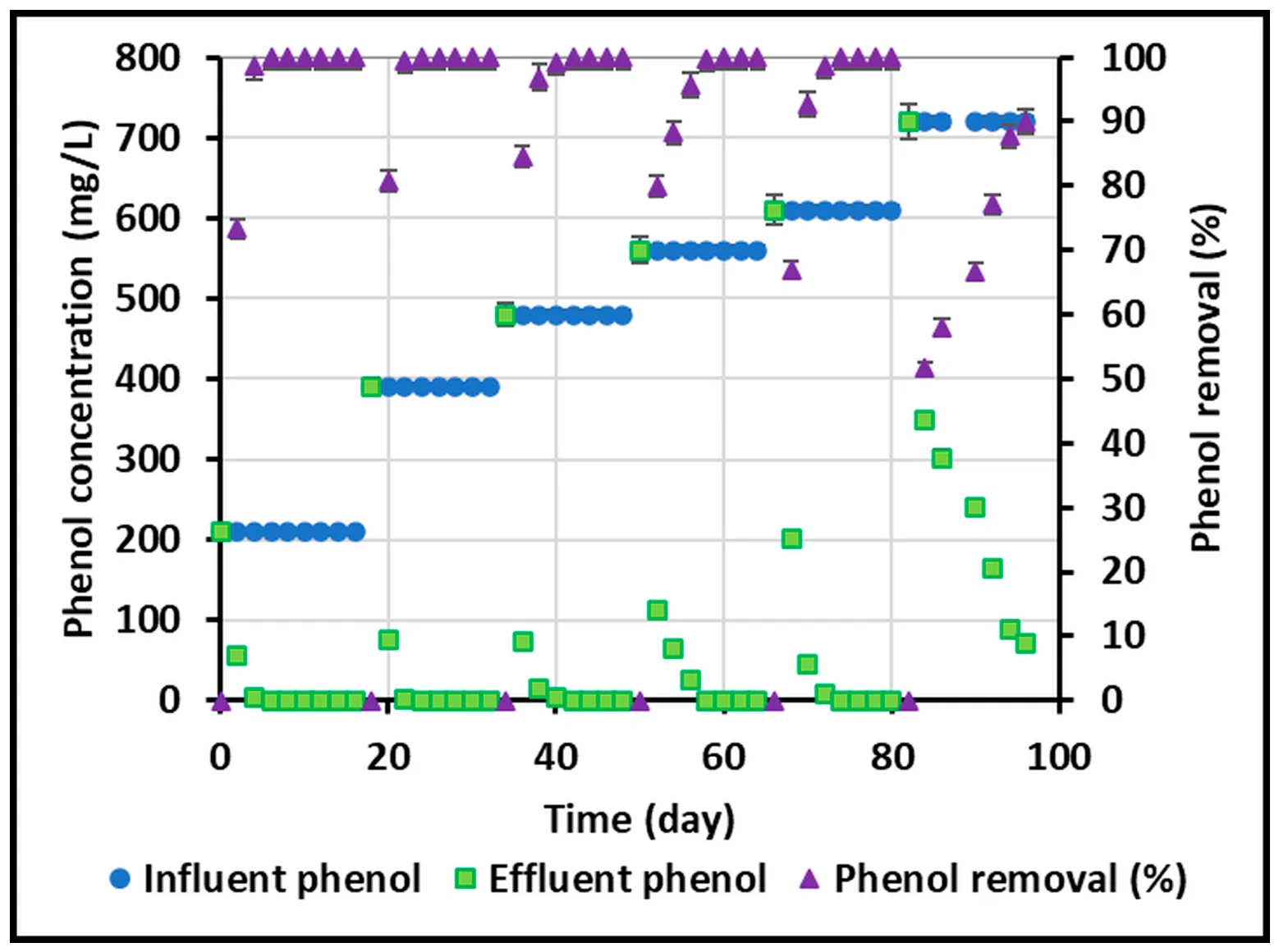
Phenol removal efficiency. Data are presented as mean ± SD of triplicate analytical measurements from duplicate experimental runs. Statistical differences were determined by one-way ANOVA; (*p* < 0.001).

Phenol elimination in AC-MFC was analyzed using High-Performance Liquid Chromatography (HPLC) across various OLs. Approximately 99% of phenol was removed within 4 days at OLs of 1.2 and 2.5 gCOD/L (Figure 3). More than 95% removal efficiency was observed within 6 days at OLs of 3.2 and 4.8 gCOD/L. At the optimal OL 6.2 gCOD/L, complete phenol removal was achieved in 8 days. However, when the OL was increased to 8.6 gCOD/L, phenol reduction efficiency dropped to 90%, likely due to decreased bacterial activity caused by high substrate concentrations and saline stress. Yaqoob et al. [34] reported phenol degradation in MFC potently enhanced the electricity generation and current density. Ziaedini et al. [35] exhibited 72% of phenol removal and 302 mV of bioelectricity generation in an air-cathode MFC operated with phenol-contaminated wastewater. A study by Cheng and Ma [36] using 5% salinity in MFC with phenol wastewater was able to achieve a maximum degradation of 54% at 220 mg/L concentration, and a further increase in the phenol concentration revealed a decline in the removal rate to 34%. Apollon et al. [37] stated organic derivatives in sediments act as an eminent source for high-power generation. Compared with previous studies, the present study demonstrated almost complete phenol degradation by the consortium, along with enhanced bioelectricity generation in the MFC operated at 6% salinity. Although phenol removal was quantitatively monitored by HPLC, specific metabolic intermediates formed during phenol biodegradation were not identified or quantified in the present study. Therefore, further metabolite profiling is required to elucidate the detailed phenol biodegradation pathways and underlying mechanisms.

### 3.3. Bioelectricity Generation

**Figure 4:**
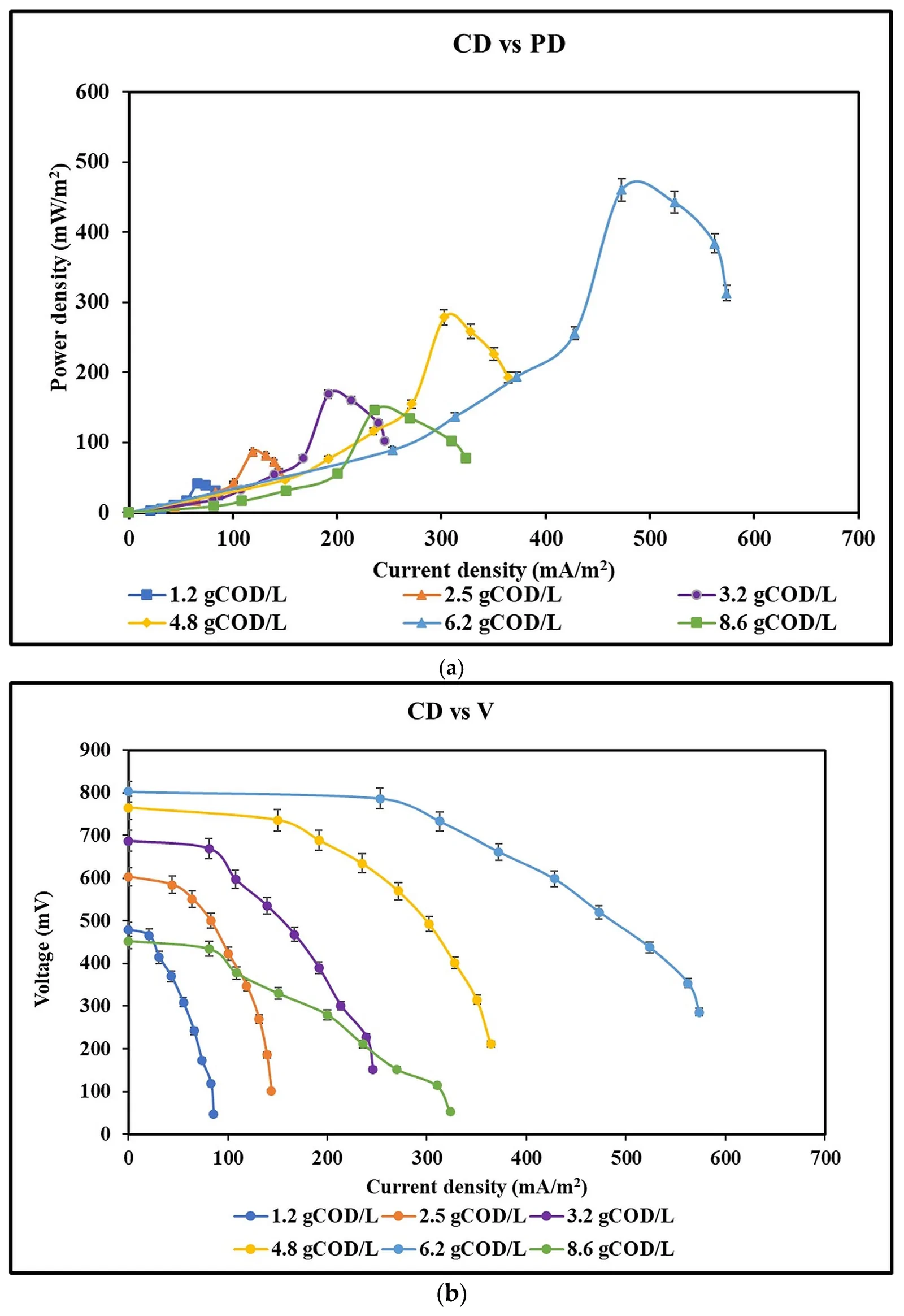
(**a**). Current density vs. Voltage in AC-MFC. Data are presented as mean ± SD of triplicate analytical measurements from duplicate experimental runs. Statistical differences were determined by one-way ANOVA; (*p* < 0.001). (**b**). Current density vs. Power density in AC-MFC. Data are presented as mean ± SD of triplicate analytical measurements from duplicate experimental runs. Statistical differences were determined by one-way ANOVA; (*p* < 0.001).

Bioelectricity generation in the MFC was strongly influenced by the wastewater organic load. The phenol-contaminated industrial wastewater was applied through a single-chamber AC-MFC. For 16 days, this operation was conducted in open-circuit mode. This open circuit helps to stabilize the microorganisms since it needs a period of time to adapt to the new environment. Then, the circuit was closed, and the air-cathode MFC was fed at different OLs. During this time, the microorganisms in the air-cathode MFC (AC-MFC) produced a constant low voltage due to the pause in biomass growth on the surface of the anode and within the anode chamber [38]. At first, the microorganisms adjusted to the new environmental conditions, leading to low-power output. As they became fully acclimatized, they began to efficiently use the organic substrate, which increased bioelectricity generation. During the industrial wastewater treatment in an AC-MFC, the polarization curve was obtained (Figure 4a,b). The external resistance range of 16,000 to 100 Ω was selected to create the polarization curves. The higher resistances stabilized quickly once the external load was removed, whereas the lower resistances stabilized more slowly, which is common behavior for fuel cells. After the 16-day open-circuit acclimatization period, the MFC was operated under closed-circuit conditions at each organic load, and the reported voltage values were measured under the respective external loads. At 1.2 gCOD/L, the voltage produced by the MFC was 480 mV, with a power density of 41.1 mW/m^2^ and a current density of 85.7 mA/m^2^. As the OL increased to 2.5 gCOD/L, the voltage rose to 604 mV, the power density increased to 86.86 mW/m^2^, and the current density reached 143.8 mA/m^2^. At 3.2 gCOD/L, the voltage increased further to 688 mV, with a power density of 169 mW/m^2^ and a current density of 245.7 mA/m^2^. As the load increased to 4.8 gCOD/L, the voltage was 765 mV, the power density was 278.7 mW/m^2^ and the current density reached 364 mA/m^2^.

The peak performance was observed at 6.2 gCOD/L, where the voltage reached 803 mV, the power density peaked at 460.6 mW/m^2^ and the current density was 573.6 mA/m^2^, attributed to efficient wastewater utilization by the microbial inoculum. The maximum bioelectricity generation at optimized OL 6.2 gCOD/L was due to the vast surface area of the carbon brush enhancing the biofilm with strong bonding, which correspondingly generates a high amount of bioelectricity. The air-cathode MFC possesses major advantages, such as reduction of oxygen at the cathode, high power yield and low internal resistance [25,26]. However, at 8.6 gCOD/L, both voltage and power density dropped significantly to 453 mV and 146.6 mW/m^2^, respectively, while the current density decreased to 323.6 mA/m^2^, indicating reduced system efficiency at higher loads. The reduction in bioelectricity generation is mainly due to the overload of substrate concentration and saturation of the electrode, which limit electron/proton transfer and the inhibition of microbial activity in MFC [39].

### 3.4. Coulombic Efficiency of the MFC

**Figure 5:**
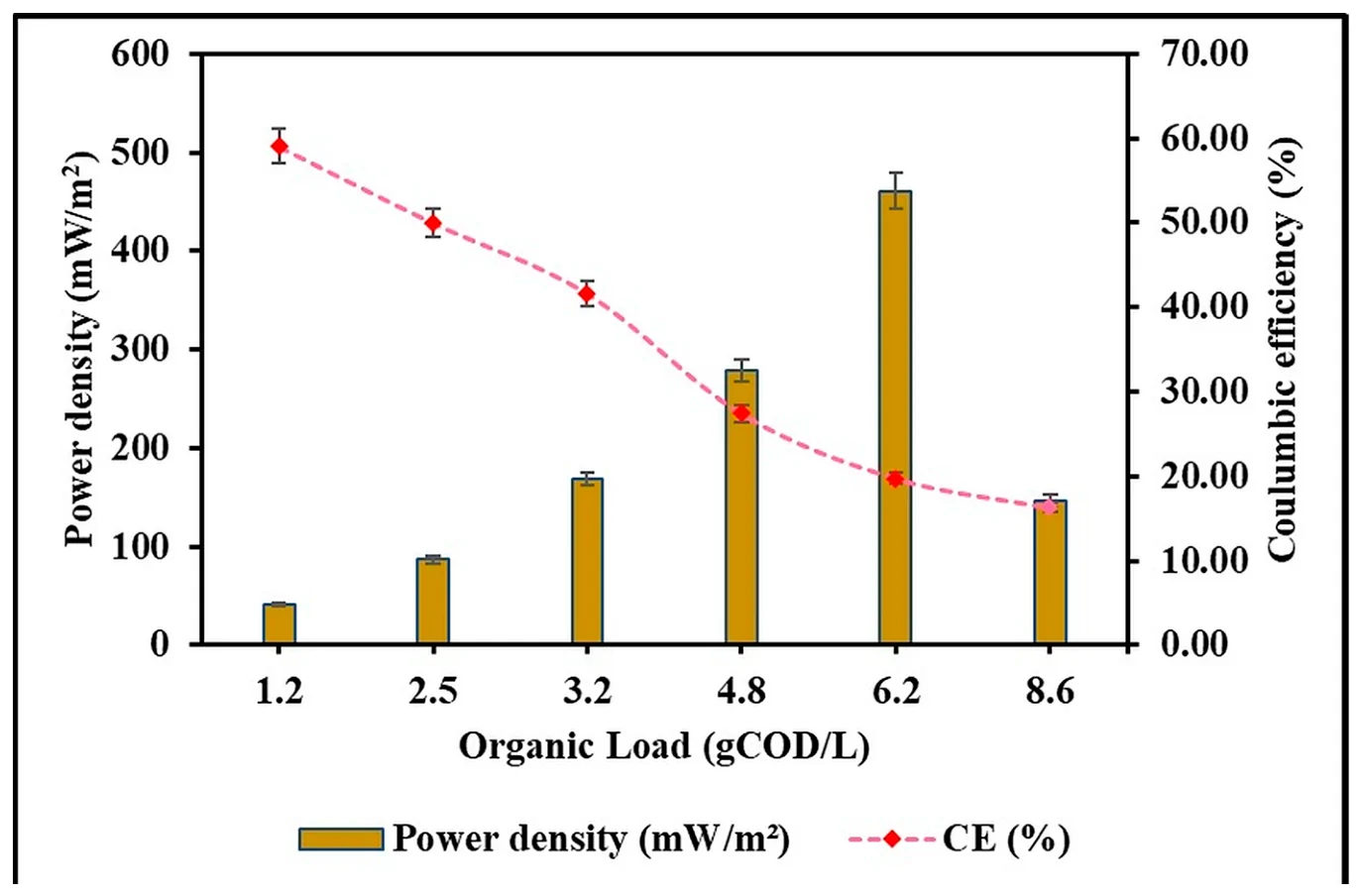
Coulombic efficiency in AC-MFC. Data are presented as mean ± SD of triplicate analytical measurements from duplicate experimental runs. Statistical differences were determined by one-way ANOVA; (*p* < 0.001).

As shown in Figure 5, the coulombic efficiency was 59.04% at a lower OL of 1.2 gCOD/L. Low organic load improved substrate consumption, resulting in higher coulombic efficiency. However, the coulombic efficiency declined to 16.26% at an OL of 8.6 gCOD/L. This drop in efficiency is likely due to competition between electrogenic bacteria and other microorganisms. The minimum coulombic efficiency was attained at 8.6 gCOD/L, with a value of 16.26% reported. Coulombic efficiency reduction in AC-MFC is due to the inhibition of electrogens activity and the increase in the diffusivity of oxygen and the substrate [25,40].

### 3.5. Bacterial Community Occupancy Profile

Occupancy of the bacterial strains in the anode and sludge region was analyzed using a high-throughput sequencing method. The results exhibited predominant occupancy of *Ochrobactrum*, *Marinobacter*, *Bacillus* and *Stenotrophomonas,* both in the anode and sludge area of the reactor operated at different organic loads (Table 1). *Rhodococcus*, *Pseudomonas* and *Acinetobacter* were present at a <10% occupancy rate in AC-MFC. Strains such as *Sphingomonas*, *Sedimentibacter*, *Alicyclobacillus* and *Microbacterium* were present with an occupancy rate of <5%. Operation of AC-MFC under optimized load of 6.2 gCOD/L revealed occupancy of *Ochrobactrum* (46.1%), *Marinobacter* (30.5%), *Bacillus* (9.2%) and *Stenotrophomonas* (7%). Previous research studies by Ghorab et al. [21] and Pugazhendi et al. [12,22] have already detailed the treatment and bioelectricity generation efficiency. The bacterial strains *Ochrobactrum* [22], *Marinobacter* [41], *Bacillus* [42] and *Stenotrophomonas* [43] are reported to degrade phenol under saline conditions. The predominant genera, including *Ochrobactrum*, *Marinobacter*, *Bacillus* and *Stenotrophomonas*, constituted important members of the microbial community under saline conditions. Some members of these genera have previously been associated with saline environments and bioelectrochemical processes; however, their direct contribution to phenol degradation or extracellular electron transfer cannot be confirmed solely from their relative abundance. Therefore, these taxa are considered potential functional contributors to the observed MFC performance rather than directly demonstrated electroactive or phenol-degrading microorganisms.

Furthermore, it is suggested that future research should focus on conducting pilot-scale studies involving extremophiles. This approach holds the potential to advance the technology for treating highly saline industrial wastewater, offering a more effective solution [44]. By addressing these areas of exploration, the field of MFCs can be further developed, paving the way for their wider application and impact in various industries.

### 3.6. AI Comparison with Experimental Data

The Random Forest electrical (power density) model demonstrated strong predictive performance (test R^2^ = 0.826, test MAE = 36.9 mW/m^2^; training R^2^ = 0.979, indicating no substantial overfitting), confirmed by 5-fold cross-validation (R^2^ = 0.803 ± 0.049, MAE = 32.3 ± 5.5 mW/m^2^, RMSE = 46.6 ± 10.1 mW/m^2^) and outperforming both the linear regression (test R^2^ = 0.368) and second-degree polynomial regression (test R^2^ = 0.722) baselines. Analysis showed that OL contributed 52.7% and operational day 47.3% to prediction strength, indicating that substrate availability and biofilm maturation contributed almost equally to bioelectricity generation.

The TCOD removal model achieved strong predictive accuracy (test R^2^ = 0.899, test MAE = 5.8%; training R^2^ = 0.983), confirmed by 5-fold cross-validation (R^2^ = 0.897 ± 0.034, MAE = 5.7 ± 1.4%, RMSE = 6.8 ± 1.8%) and outperforming the linear baseline (test R^2^ = 0.694), while the second-degree polynomial model achieved a slightly higher test R^2^ (0.924, MAE = 4.49%), reflecting smooth saturation kinetics. Operational day contributed 71.2% to predictive strength, indicating biofilm maturation as the dominant factor influencing treatment efficiency, while OL contributed 28.8%.

**Figure 6:**
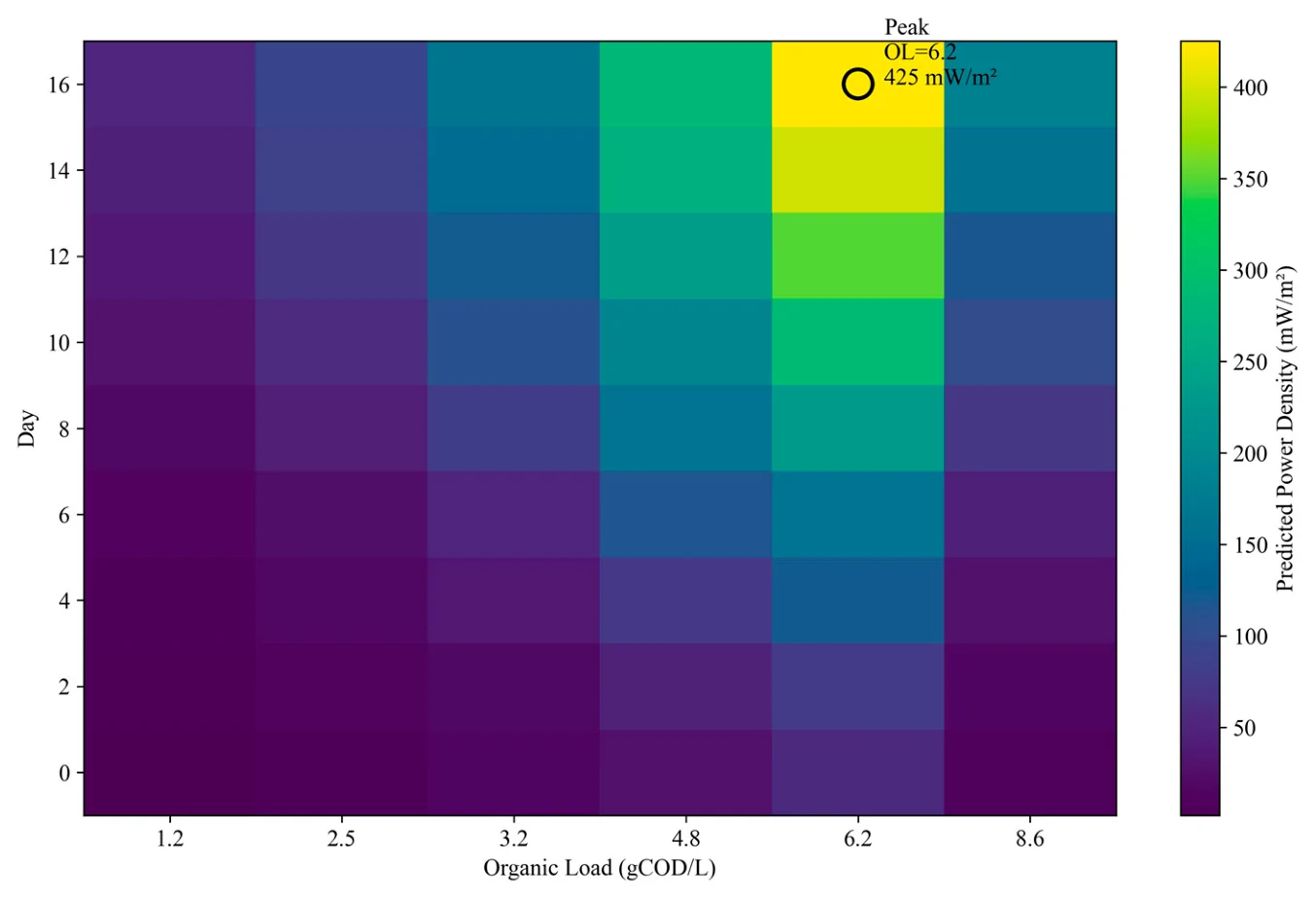
Random Forest-predicted power density (mW/m^2^) across operational day and organic load (OL). The marked point indicates the model-predicted peak.

**Figure 7:**
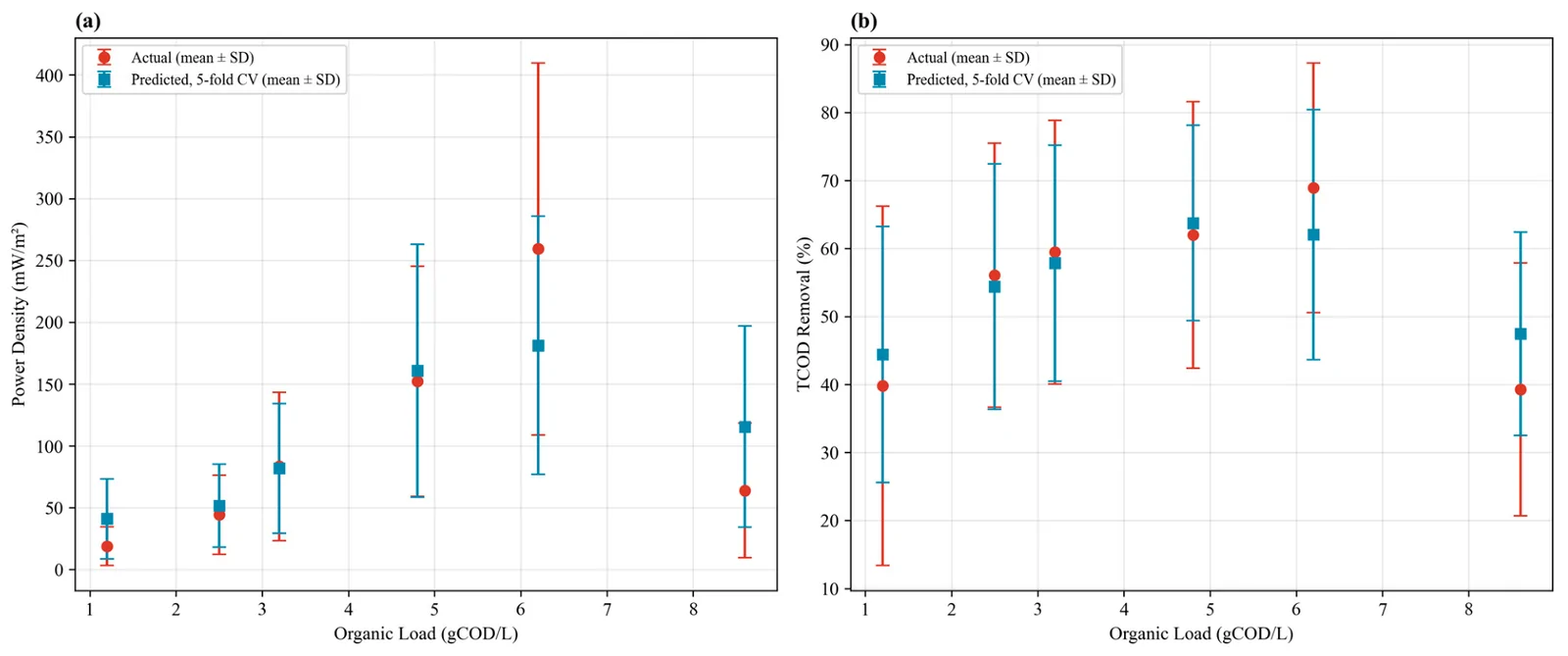
Actual versus 5-fold cross-validated-predicted values (mean ± SD per OL stage) for (**a**) power density and (**b**) TCOD removal.

Figure 6 shows the Random Forest-predicted power density surface across operational day and OL, with the model-predicted peak located at 6.2 gCOD/L. Figure 7 shows the actual versus 5-fold cross-validated-predicted values (mean ± SD per OL stage) for power density and TCOD removal, illustrating the reliability of both models across the tested range of organic loads.

Multi-objective optimization integrating normalized predicted power density and TCOD removal (Table 2) identified 6.2 gCOD/L as both the highest-ranking operational condition (combined score = 1.00) and the condition producing the highest predicted electrical output (~407 mW/m^2^), consistent with the experimentally validated optimum reported for this system (460.6 mW/m^2^ measured power density, 90% TCOD removal). The lowest-ranked condition, 8.6 gCOD/L, combined a comparatively high predicted power density with the lowest predicted TCOD removal of all stages tested, reflecting the trade-off between electrical output and treatment efficiency at excessive organic loads.

Model comparison against baseline regressors revealed target-dependent performance. For power density, the Random Forest model outperformed both baseline comparators (test R^2^ = 0.826 vs. 0.722 for second-degree polynomial regression and 0.368 for multiple linear regression), highlighting the nonlinear, non-monotonic relationship between substrate loading and electrical yield. For TCOD removal, the second-degree polynomial model yielded a slightly higher test R^2^ (0.924, MAE = 4.49%) than Random Forest (0.899, MAE = 5.78%), indicating that organic degradation efficiency followed a relatively smooth parabolic trajectory across loading stages. Random Forest was nonetheless retained for the integrated evaluation to maintain methodological consistency across both targets and to exploit non-parametric Gini feature importance. This is consistent with the well-documented behavior of air-cathode MFCs, in which electrochemical activity improves with increasing organic load up to a threshold before declining sharply due to activation losses, ohmic losses, and competition between electrogenic and non-electrogenic populations at excessive loading [18,29].

The feature importance results provide mechanistic insight consistent with the underlying biology of the system. For power density, OL and operational day contributed almost equally (52.7% and 47.3%, respectively), suggesting that instantaneous substrate availability and the progressive maturation of the electroactive biofilm jointly govern electron transfer and current generation. In contrast, operational day dominated the TCOD removal model (71.2% versus 28.8% for OL), indicating that treatment efficiency in this system is primarily driven by biofilm development and community establishment over the course of each loading stage rather than by the organic load itself. This is in line with prior reports that thicker, more established biofilms on high-surface-area electrodes accelerate substrate mineralization independent of loading regime [26].

Notably, the multi-objective optimization identified 6.2 gCOD/L as the top-ranked condition by combined score and simultaneously as the condition with the highest predicted power density, in full agreement with the experimentally determined optimum for this reactor (maximum voltage, power density, and TCOD/SCOD removal all observed at 6.2 gCOD/L). This convergence between a purely data-driven, cross-validated model and the independently measured wet-laboratory optimum strengthens confidence that the Random Forest framework captured genuine operational relationships rather than sampling artefacts and supports its use as a decision-support tool for organic load selection in halophile-augmented air-cathode MFCs treating saline industrial wastewater. The comparatively low combined score at 8.6 gCOD/L, despite a moderate predicted power density, reflects the steep decline in treatment performance at this stage and is consistent with reports that excessive organic loading inhibits the electrogenic activity of the strains and substrate/electrode saturation, thereby limiting electron and proton transfer [25,29]. To ensure that the identified optimum was not sensitive to the equal-weighting scheme (0.5 × PD_norm + 0.5 × Removal_norm), a sensitivity analysis was performed by varying the power density weight w from 0.0 (treatment-only focus) to 1.0 (energy-only focus). Because the 6.2 gCOD/L stage simultaneously achieved both the highest predicted steady-state power density (407.5 mW/m^2^, PD_norm = 1.00) and the highest stable TCOD removal (85.4%, Removal_norm = 1.00), it represented a strictly Pareto-dominant state. Consequently, the ranking of 6.2 gCOD/L as the primary operational optimum was completely robust and invariant across the entire weighting continuum.

Several limitations should be noted. The dataset, while sufficient for robust cross-validated estimates given the constrained experimental design (six OL stages, single reactor replicate per stage), remains small by machine learning standards, and the reported cross-validation standard deviations indicate non-trivial fold-to-fold variability, particularly for power density at higher organic loads. Future work should incorporate additional operational replicates and, where available, microbial community composition as model features, given the documented role of dominant genera such as *Ochrobactrum*, *Marinobacter* and *Bacillus* in phenol degradation and electron transfer under saline conditions [40,41]. Extending this artificial intelligence technology (AIT) framework to real-time or online prediction, and validating it across independent reactor configurations, would further support its translation into practical process-control tools for the scale-up of AC-MFC technology for industrial saline wastewater treatment [26]. Microbial growth and biomass development were not directly quantified in this study; therefore, the relationship between biomass development, pollutant removal, and bioelectricity generation could not be quantitatively established. Future studies should include direct biomass measurements to better elucidate these relationships. Based on our previous study, yeast extract supplementation enhanced bacterial growth under saline conditions, thereby improving biodegradation efficiency [45]. Future studies could, therefore, evaluate yeast extract supplementation in the present halophilic AC-MFC system to further enhance treatment performance and bioelectricity generation.

From a broader process development perspective, translating microbial electrochemical systems from laboratory-scale experiments into practical wastewater treatment applications requires careful consideration of reactor configuration, electrode and separator selection, mass transfer, long-term operational stability, real-substrate variability, and system-scale operation. Recent reviews have emphasized that component selection and reactor engineering remain important challenges in achieving reliable performance with real wastewater and advancing toward larger-scale implementation [46]. In addition, recent work on microbial electrolysis systems treating waste-derived substrates has highlighted the importance of integrating wastewater treatment with energy recovery while accounting for substrate complexity, process configuration, and scale-up constraints [47]. Although the present study demonstrates promising treatment performance and electricity generation under saline conditions, further validation using independent reactors, extended operating periods, continuous-flow configurations, and wastewater streams with variable composition is necessary before practical scale-up can be adequately assessed.

To facilitate the future application of microbial fuel cells (MFCs), further research should focus on improving their performance, durability, and scalability. In particular, the development and evaluation of biocompatible, high-surface-area electrode materials may enhance biofilm adhesion and support the long-term activity of electroactive microorganisms, thereby contributing to stable bioelectricity generation. In parallel, the optimization of reactor configuration, substrate flow rate, hydraulic retention time (HRT), and electrode architecture is important for maintaining consistent treatment performance under different operating conditions. Carbon-based electrodes remain widely attractive because of their biocompatibility, electrical conductivity, chemical stability, and relatively low cost [48]. However, their long-term performance and economic feasibility under practical operating conditions require further investigation. Moreover, the scalability of MFCs for applications such as wastewater treatment plants and remote power generation should be systematically evaluated, considering energy recovery, operational stability, maintenance requirements, and overall process economics. Further development of cost-effective and integrated MFC technologies could contribute to sustainable wastewater treatment and resource recovery [29,49,50].

## 4. Conclusions

An AC-MFC operated at various organic loads (1.2, 2.5, 3.2, 4.8, 6.2, and 8.6 gCOD/L) under saline conditions (6%) was examined to study treatment efficiency and bioelectricity generation. Among the various load ranges tested in the AC-MFC, the optimal performance was observed at a load of 6.2 gCOD/L. Total COD (90%), soluble COD (89%), TSS (67%) and complete removal phenol within 8 days were achieved at optimized organic loads to prove the saline industrial wastewater treatment efficiency. In the optimized condition, the maximal bioelectricity generation of 803 mV was obtained with corresponding power density (460.6 mW/m^2^) and current density (573.6 mA/m^2^), respectively. The dominant halophilic bacterial consortium in the AC-MFC was composed of highly robust strains (*Ochrobactrum, Marinobacter* and *Bacillus*) to treat the saline wastewater integrated with bioelectricity generation. The AIT-based Random Forest model also predicted 6.2 gCOD/L as the optimized organic load for high PSIWW treatment efficiency with corresponding bioelectricity generation. These results demonstrate the effectiveness of the dominant halophilic bacteria in treating phenol-contaminated industrial saline wastewater and generating bioelectricity in AC-MFC. This study advocates the use of AC-MFC integrated with inoculation of halophilic consortium as an eco-friendly approach for industrial wastewater treatment and bioelectricity generation. Future research should investigate the long-term operational stability and scale-up potential of the AC-MFC system, while further validating the AI model using saline industrial wastewaters with different compositions and operating conditions to assess its broader practical applicability.

## Figures and Tables

**Table 1:** Bacterial community analysis of anodic and sludge region samples at different OLs in AC-MFC.

Organic Load (gCOD/L)	Bacterial Community—Anode (%)						Bacterial Community—Sludge (%)						Accession Number
	OL1.2	OL2.5	OL3.2	OL4.8	OL6.2	OL8.6	OL1.2	OL2.5	OL3.2	OL4.8	OL6.2	OL8.6	
Bacterial Strain													
***Ochrobactrum***	**26.4**	**29.8**	**34.6**	**38.5**	**46.1**	**34**	**23.4**	**25.6**	**28**	**30.6**	**34.7**	**29.6**	**PV689948**
***Marinobacter***	**17.3**	**19.4**	**22.2**	**25.8**	**30.5**	**25.2**	**16.5**	**17.7**	**18.5**	**19.4**	**22**	**19.8**	**PV689949**
***Bacillus***	**13.6**	**12.3**	**11.2**	**10**	**9.2**	**11.5**	**15.5**	**15.8**	**16.8**	**17.5**	**18**	**16.2**	**PV689950**
***Stenotrophomonas***	**11.2**	**10.4**	**9**	**8.2**	**7**	**9**	**14.5**	**15**	**15.7**	**16.3**	**16.7**	**15.5**	**PV689951**
*Rhodococcus*	9	8.5	7.1	6	4	6.5	9.1	8	7	6	4	6.6	PV689952
*Pseudomonas*	8.5	7.7	6	5	2	5.4	8	7.5	6.5	5.2	2	5	PV689953
*Acinetobacter*	7	6.5	5.5	4	1	4.8	6	5.4	4.5	3	1.6	4	PV689954
*Sphingomonas*	4	3.4	2.8	1.5	0.2	2.6	3	2.5	2	1	1	2.3	PV689955
*Sedimentibacter*	2.5	2	1.6	1	0	1	2	1.5	1	1	0	1	PV689956
*Alicyclobacillus*	0.5	0	0	0	0	0	1	0.5	0	0	0	0	PV689957
*Microbacterium*	0	0	0	0	0	0	1	0.5	0	0	0	0	PV689958

Note: Bold formatting indicates the dominant bacterial strains in the halophilic consortium at different OL.

**Table 2:** Machine learning-based performance summary across organic load conditions.

OL (gCOD/L)	Predicted PD, Last Day (mW/m^2^)	Predicted TCOD Removal (%)	Normalized PD	Normalized Removal	Combined Score	Rank
6.2	407.5	85.4	1.00	1.00	1.00	1
4.8	279.7	81.7	0.63	0.87	0.75	2
3.2	157.9	77.4	0.28	0.72	0.50	3
2.5	100.4	72.6	0.11	0.55	0.33	4
1.2	60.6	67.8	0.00	0.38	0.19	5
8.6	189.1	56.9	0.37	0.00	0.19	6

## Data Availability

The original contributions presented in this study are included in the article. Further inquiries can be directed to the corresponding author.

## Abbreviations

The following abbreviations are used in this manuscript:

AC-MFC	Air-Cathode Microbial Fuel Cell
AI	Artificial Intelligence
AIT	Artificial Intelligence Technology
ANOVA	Analysis of Variance
BLAST	Basic Logarithmic Assign Search Tool
CE	Coulombic Efficiency
COD	Chemical Oxygen Demand
CV	Cross-Validation
gCOD/L	gram chemical oxygen demand per liter
HRT	hydraulic retention time
HPLC	High-Performance Liquid Chromatography
mA/m^2^	milliamperes per square meter
MAE	Mean Absolute Error
MFC	Microbial Fuel Cells
mV	milli Volt
mW/m^2^	milli watts per meter square
NCBI	National Center for Biotechnology Information
OL	Organic Load
PSIWW	Phenol-Contaminated Saline Industrial Wastewater
PD	Power Density
RMSE	Root Mean Squared Error
SCOD	Soluble Chemical Oxygen Demand
SD	Standard Deviation
SPSS	Statistical Package for the Social Sciences
TCOD	Total Chemical Oxygen Demand
TSS	Total Suspended Solids
